# Patient Living With Chronic Illness Perception of Interprofessional Collaboration in a Telehealth Context in Primary Care: Protocol for a Qualitative Descriptive Study

**DOI:** 10.2196/79019

**Published:** 2025-12-24

**Authors:** Monica McGraw, Isabelle Gaboury, Yves Couturier, Marie-Dominique Poirier, Marie-Eve Poitras

**Affiliations:** 1 Department of Family Medicine and Emergency Medicine, Faculty of Medicine and Health Science, Université de Sherbrooke, 2500, Boul de l'université Sherbrooke, Saguenay, QC, J1K 2R1, Canada, 1 819-821-8000; 2 Research Team on Optimal Professional Practices in Primary Care, Saguenay, QC, Canada; 3 Department of Family Medicine and Emergency Medicine, Université de Sherbrooke, Longueil, QC, Canada; 4 Department of Social Work, Faculty of Letters and Human Sciences, Université de Sherbrooke, Québec, QC, Canada

**Keywords:** primary care, interprofessional collaboration, chronic disease, telehealth, patient engagement, qualitative method

## Abstract

**Background:**

The enhancement of primary care and the prevalence of chronic diseases are key issues worldwide, especially in Canada. The rising incidence of chronic illnesses, now the leading cause of mortality worldwide, creates complex challenges that can compromise the quality of care provided to patients. A lack of communication directly affects relational continuity. These challenges highlight the importance of establishing clear patient pathways within interprofessional teams, ensuring that information is shared efficiently and the continuity of care is coordinated effectively, especially in a telehealth context. Since 2019, telehealth has become an essential tool for patients with chronic disease, although often implemented with no specific infrastructure. Interprofessional collaboration (IPC) plays a critical role in the use of telehealth in managing chronic diseases.

**Objective:**

This study aims to understand the IPC process as experienced by patients in a telehealth context within primary care, with a focus on patient engagement. More specifically, the study’s objectives are (1) to describe the IPC process in telehealth within primary care from the perspective of patients living with chronic conditions; (2) to identify, in collaboration with patients living with chronic disease, the barriers and facilitating factors in this process; and (3) to understand the engagement of these patients in relation to the IPC process in a telehealth context.

**Methods:**

This qualitative research study is based on constructivist research methodology to describe the process of IPC in the telehealth context in primary care from the perspective of patients living with chronic disease. The research team will construct knowledge derived from the interpretation of information that was obtained during the interviews with participants. To meet the study’s objectives, we carried out qualitative journey mapping for data collection. Individual interviews were analyzed iteratively. This method is useful for this research as it visually and collaboratively captures patients’ lived experiences.

**Results:**

Data collection was completed in November 2024. A total of 22 interviews were conducted. The project was funded in March 2022. As of December 2025, all participants had been recruited, and the qualitative data analysis was currently underway. Multiple manuscripts are in development, and the first set of findings was submitted for publication in fall 2025.

**Conclusions:**

The results of this study will support and improve the IPC process in the telehealth context by providing concrete insights into patients’ experiences, identifying gaps and strengths in current collaborative practices, and offering evidence-based recommendations. Journey mapping will help identify potential facilitating factors for improving primary care in the telehealth context according to the patient’s journey. The results will be used to build a practical guide (in phase 2) supporting IPC in the primary care telehealth context.

## Introduction

Chronic disease is recognized as a long-term, persistent, and mostly noncommunicable condition requiring ongoing management [1]. In Canada, 44% of adults aged 20 years and older live with chronic diseases [2]. This percentage increases significantly with age [2]. Over 4.9 million (73%) Canadians aged older than 65 years live with at least one chronic disease. For patients living with these diseases, access to primary care is an essential element contributing to their continuity of care and patient engagement [1,3,4]. However, the lack of access to primary care does not promote efficient chronic disease management or active engagement of patients living with chronic disease [5,6] and ultimately affects their health [7,8]. The COVID-19 pandemic has amplified the delay in accessing primary care [9,10].

Various publications describe patients living with chronic disease as those who consult primary care most frequently [1,11]. These patients receive regular interprofessional follow-up by the primary care team, including nurses, family doctors, pharmacists, social workers, and others [12,13]. This interprofessional necessity of chronic disease management and the need to provide optimal interprofessional care implies that each professional involved must apply the recommended interprofessional practice standards [14,15]. Interprofessional collaboration (IPC) is defined as the process by which professionals from different disciplines develop modalities of practice that provide a coherent, integrated response to the needs of the individual, their loved ones, and the community [14]. To ensure continuity of care, it is essential that all professionals contribute to a shared, coordinated approach that supports seamless transitions and consistent communication throughout the patient’s care trajectory [16-18].

During a well-established IPC process, a way to improve the quality of care is to offer care with the support of telehealth to increase access [19-21]. Telehealth is defined as “any interaction between a patient and a health care professional that takes place at a distance and uses some form of information or communication technology (ie, telephone, email, SMS, videoconferencing platform, electronic medical record [EMR])” [22]. In several Canadian provinces, telehealth is being used to better meet patient needs, such as improving accessibility to care [6,23]. Telehealth experienced significant growth during the SARS-CoV-2 pandemic but has since lost momentum. However, some studies point out that Canadians want to maintain access to telehealth consultations in the postpandemic context, mainly because of the time saved on travel and improved access to an appointment [24]. Telehealth consultations not only increase accessibility but also reduce the costs associated with care for the health care system when used optimally and appropriately [25,26]. For health care professionals and the health care system, proper use of telehealth facilitates communication between health care professionals, which ultimately supports IPC [4,20,27]. Despite the benefits of offering care through telehealth, it can have negative effects on patient health if used suboptimally or inappropriately [19,20], affecting IPC and the quality of patient engagement [24,28].

This acceleration was not only because of the pandemic [29,30] but also to support access to care in certain more remote regions [31]. Telehealth is supported in the literature for improving access to care and helping the health system adapt to demographic changes [6,20,32,33]. Although telehealth offers many advantages and is appreciated by patients and professionals alike [34,35], some studies point to the importance of further adapting this service modality to the needs of patients living with chronic diseases (eg, video-assisted telehealth for older patients due to digital literacy or patients with technological challenges). Other studies emphasize that to achieve positive effects for patients, it is essential to support professionals and patients in their appropriation of this intervention modality and to promote the maintenance of IPC despite the remote work or care context [36]. This might be explained in part because telehealth is often used without a defined framework and left to the discretion of each care setting [6,17,36]. Telehealth implemented without a clear framework (tools, work procedures, etc) for primary care increases work overload and stress for professionals [13,27,30]. Including the perspective of patients living with chronic diseases remains crucial and essential to guide professionals in keeping telehealth and IPC responsive to their needs [15,20].

The patient perspective helps improve health outcomes for individuals and increases patient satisfaction, confidence, and engagement in their health [37]. However, to date, the patient perspective of the IPC process, including patient engagement among patients living with chronic disease in a telehealth context, is still underresearched [38,39]. The Montreal model (Multimedia Appendix 1), the Canadian Institutes of Health Research, and the Strategy for Patient-Oriented Research also support this approach to integrating patient partners into the research team [40-42].

This study will aim to understand patients’ engagement experience in the process of IPC in a primary care telehealth setting, considering the patient perspective. To achieve this aim, this research project has three specific objectives:

1. To describe the telehealth IPC process in primary care from the perspective of patients living with chronic disease
2. To identify, in collaboration with patients living with chronic disease, the barriers and facilitators in this process
3. To understand how these patients are engaged in the IPC process in a telehealth context.

## Methods

### Overview

The focus of the study is to better understand patient perspective on telehealth. This study therefore includes one patient partner in the research team. Meetings were held with the patient partner and the research team to develop the study protocol, interview guide, and mapping template, laying the groundwork for future data analysis and ensuring that findings can be effectively translated into practice through knowledge transfer strategies. This approach is based on the recommendations of Pomey et al [40] and their conceptual framework for patient engagement in research.

This qualitative research is based on a constructivist research methodology, where the research team constructs knowledge from the analysis of information obtained during dialogues with participants [43,44]. To meet the study’s objectives, we carried out a qualitative journey mapping data collection, following the approach of Trebble et al [45]. This method enables users (in this case, patients living with chronic diseases) to be involved early and throughout the mapping development process. Pathway mapping in telehealth (Multimedia Appendix 2) allows us to delineate the patient journey, detailing their engagement and experiences with telehealth services at the primary care level. This approach helps identify areas for enhancing the quality of patient care. Journey mapping also places the patient at the center of analysis to better understand and improve the process of IPC [46]. It also allows us to identify the points of contact between patients and professionals during the telehealth consultation process (ie, method of communication, pathway to an appointment, and other processes) based on patients’ expressed or unexpressed needs [45]. This type of approach provides a better understanding of elements starting from service entry, navigation, and ongoing experience right through to patient discharge from the health care system [46].

Two conceptual frameworks will be used to structure this study. To enrich the understanding of patient engagement regarding the IPC process, the IPC-related domains of the Interprofessional Education for Collaborative Patient-Centred Practice (IECPCP) model (Multimedia Appendix 3) will be used for this project [14]. This model of IPC is patient centered and intends to improve patient outcomes. In this model, the process is divided into four dimensions: (1) governance; (2) rules to structure the team; (3) shared goals and vision; and (4) sense of belonging [14].

To adequately integrate the dimension of patient-centered care and to focus specifically on patient engagement within care processes, the Montreal model [40] can be used as a conceptual reference. Although this model primarily emphasizes the concept of partnership rather than engagement per se, it nonetheless offers a useful framework for conceptualizing varying levels of patient engagement. For the purposes of this project, only the dimension related to patient engagement within care processes will be retained, allowing for a targeted application of the model in the context of IPC and chronic disease management.

The patient’s voice is considered on an equal footing with other stakeholders in the primary health care team. Moreover, the patient can also be supported by a caregiver to better express their experience [40]. Ultimately, this model relies on patients’ experiential knowledge, enabling them to make informed decisions and exercise leadership at the same level as health care professionals [40]. The direct care delivery section is a logical level of the model that refers to interactions between professionals, as individuals and as a team, and patients [40]. It provides an understanding of the level of engagement of professionals and patients at the clinical level. In addition, the telehealth context will be highlighted in the consultation section of the Montreal model, where the patient must have had a telehealth consultation [40]. Finally, the components present in this model will support our understanding of the phenomenon of patient engagement living with chronic diseases in a telehealth context.

### Study Population

The target population is all adult patients living with chronic diseases who have consulted virtually through telehealth in a primary care clinic in which different health care professionals practice collaboratively (ie, the presence of communication channels or an alternating care plan that is already established).

The settings studied for this research will be university primary care clinics called “university family medicine groups” (U-FMGs) in Quebec. U-FMGs (groupings of family physicians who work together and in close collaboration with other health and social services professionals) [47] are the main model of primary care service in Quebec [13,48]. Some family medicine groups are affiliated with universities and integrate the training of family medicine residents and externs. This study will focus on U-FMGs that practice telehealth.

### Sampling and Recruitment Strategy

We will use purposive sampling to provide a diverse description of telehealth IPC experiences in primary care [49,50]. This type of sampling allows the research team to recruit participants who meet specific inclusion criteria and are representative of the phenomenon under study [50]. We will strategically select research sites and sampling methods based on their perceived richness and utility in providing comprehensive insights into the phenomenon under investigation [51]. The literature also points to other important strengths, such as lower cost, convenience, and reduced data-collection time [52]. A 2-tiered recruitment will be used, namely the development of a partnership with U-FMGs and subsequently the direct recruitment of patients from partner U-FMGs. This partnership will enable the team to recruit 2 U-FMGs. These U-FMGs will be recruited face-to-face to enable us to develop a relationship of trust between the research team and the U-FMGs administration from the outset. From the first meeting, the team will ensure the existence of an IPC process (eg, presence of communication channels or alternating care plans already established), as well as a French- or English-speaking clientele and the presence of telehealth consultations for patients. Once the U-FMGs have been selected, the research team and U-FMG managers will work together to ensure adequate patient recruitment. Posters will be placed in waiting rooms with information relevant to the study to solicit participation from the target population. Data collection was concluded after 22 interviews once thematic saturation was achieved, that is, when no new themes or insights emerged from successive interviews, consistent with qualitative methodological guidance [53-55]. This decision was discussed and validated among the research team. Data analysis will be carried out on an ongoing basis throughout the data collection process to check for redundant information, for example, when the research team begins to hear the same comments repeatedly, using a saturation grid [54,55] as a method of keeping track of this redundancy. This will allow them to stop data collection and finalize the analysis.

As inclusion criteria, adult participants have to declare that they live with at least 2 chronic diseases that are recognized by the Public Health Agency of Canada [2], have consulted at least 3 times in the last year in the participating clinic, and have had at least one of the 3 appointments in a telehealth context. Selected patients must have consulted the primary care clinic in connection with their chronic diseases. Recruitment from this population is justified by a higher likelihood of having had IPC experiences in a telehealth setting due to the need to maintain follow-ups in primary care [23,51].

### Data Collection

We will be collecting data in the format of a journey mapping through a maximum of 90-minute semistructured interviews describing patient engagement within care processes in IPC from the perspective of patients living with chronic diseases [50,56]. These interviews will be conducted in either French or English, depending on patient preference. This type of interview allows the researcher to ask a question or several questions from a predetermined list, depending on the direction of the dialogue. This type of interview allows the patient’s experience to emerge. Due to the geographical distance between the researcher or interviewer researcher and the potential U-FMGs and for the advantage and flexibility of the patient, the interviews will be conducted using the Microsoft Teams platform [57] to maintain consistency of format between each participant.

The semistructured interview guide will be oriented toward the IPC’s experience in the engagement within care processes in the primary care clinic in the last 12 months in a telehealth context and was developed considering both conceptual frameworks [14,40]. We will begin the interview with an initial question: “Tell me about your experience with your clinic’s team of health care professionals during telehealth consultations in the last 12 months.” The purpose of this initial question is to activate memories of the past to enable the start of a conversation that will map the journey of these patients living with chronic disease and their experiences using telehealth [58,59]. The interview guide includes subquestions to gather information on the patient’s journey, as well as facilitators and barriers. The interview questionnaire will be customized as needed to capture the IPC experience of each patient living with chronic diseases in a telehealth context.

As soon as consent to this study has been obtained, all recruited participants will be asked to complete a sociodemographic form covering their clinical and professional sociodemographic characteristics as supported by the conceptual frameworks. A logbook will also be used by the first author to record her field notes.

### Data Analysis

We will analyze the individual interviews qualitatively and iteratively using the method reported by Gale et al [60]. This method is useful for analysis by different people from interprofessional teams, including those with little experience in qualitative analysis [60]. This method of analysis comprises seven steps: (1) transcription; (2) familiarization with the data; (3) coding; (4) development of the analytic framework; (5) application of the analytic framework; (6) graphical representation of the data; and (7) interpretation of the data. Analysis in this project will take the form of journey mapping, a method used to visually and thematically structure the experiences shared by patients. This approach captures the sequence of events, emotions, interactions, and decision points encountered throughout their care journey, allowing for a comprehensive understanding of how they navigate the health care system and how IPC and telehealth shape their experiences. In accordance with the principles of patient-oriented research, the analytical process is designed to balance meaningful patient engagement with methodological rigor. The patient partner’s involvement is concentrated in steps 4 and 7, where experiential knowledge is most valuable for shaping the analytic framework and interpreting findings, while earlier phases are completed by the research team to maintain consistency in data coding and ensure analytic reliability [61]. Coding will be both inductive (based on respondents’ subjective experience) and semideductive (guided by the IPC theme domains of the IECPCP model and the Montreal model) [14,40,62]. The domains of the IECPCP model are not used to generate a codebook but to guide the structure of the journey-mapping framework. Each of the 4 domains, governance, team structure, shared goals and vision, and sense of belonging, served as an organizing lens to segment the mapped data and visually represent how IPC and patient engagement unfolded across the patient’s care experience. This allowed the mapping process to remain theoretically grounded while still reflecting the participants’ lived experiences derived through inductive analysis.

The first author will do field noting during the interview process and may validate certain information with the patient during the interview [15,60]. Next, the first author will ensure that the information is fully transcribed in cartographic format by listening to the interviews (step 1). She will be responsible for familiarizing herself with the transcribed data, auditory data, and interview notes (step 2) while entering the information directly into the journey mapping. Mapping is a practical and visual method that supports reflection on continuity of care, reduction of wait times, and enhancement of patient safety, aligning with the principles of the Montreal model by fostering meaningful patient engagement and with the IECPCP model through its emphasis on interprofessional, patient-centered collaboration [45,63]. At this stage, the first author will invite a subset of participants to review the coded information for accuracy (member checking). Participants will have the opportunity to clarify or elaborate on their responses before data analysis proceeds.

The first author will then begin coding the data collected (step 3), thus initiating the mapping condensation. As the process moves forward, the analysis will be iteratively refined through journey mapping, which serves as a core analytic and visual tool to trace patient experiences across various stages of care. This method enables the research team to revisit and revise codes, frameworks, and interpretations as new insights emerge from ongoing data collection. By mapping the care trajectories of patients living with chronic disease in a telehealth context, the research maintains continuous alignment with the principles of patient engagement and IPC, thereby enhancing both the accuracy and the contextual relevance of the findings [45,60,63]. Once the data have been coded, the research team, together with the patient partner, will meet to verify (co-code) the data (step 4). The research team will then apply the analysis frameworks to the collected and coded data (step 5). Following the data condensation process, the first author will produce graphic representations in the form of journey maps (step 6), visually illustrating the key themes and pathways that emerged from the analysis. These maps will reflect the condensed data and highlight patients’ experiences, interactions, and critical moments across their care trajectories. She will be supported by one patient partner, and they will meet to finalize the analysis and interpretation of the data. At this stage, the team and patient partner will verify the data collected with the condensed mapping. Throughout the analysis, the research team will use the two conceptual frameworks to guide them. In addition, particular attention will be given to equity criteria when analyzing according to the PROGRESS+ (place of residence, race/ethnicity/culture/language, occupation, gender/sex, religion, education, socioeconomic status, and social capital plus) model [64] but not limited to this model, since these factors can influence IPC, the use of technological tools and their functions, and the delivery of telehealth [65]. PROGRESS+ is associated with diverse characteristics of the target population, ensuring their representation [64]. These criteria will be considered during the recruitment and at every stage of the project.

All barriers and facilitators related to IPC will be retained as information to be analyzed and interpreted to describe the IPC process. The team will ensure that the interview and the information retained focus directly on the experiences related to the IPC process and the patient engagement in the telehealth context. The research team will maintain reflexive journals to document assumptions, decisions, and potential biases throughout the data collection and analysis process. Reflexivity will be discussed regularly in team meetings to ensure that interpretations remain grounded in participants’ perspectives.

### Ethical Considerations

Before the study, the ethics committee at the Centre intégré Universitaire de santé et de services sociaux of Saguenay-Lac-Saint-Jean acted as the reviewer and granted ethics approval on May 9, 2024 (2023‐047). The project was also approved by the Ethics Committee of the Université de Sherbrooke’s Faculty of Education and Social Sciences on December 15, 2023 (2023‐4057) and by the Vitalité Health Network on January 23, 2024 (101931). Written consent will be obtained from all study participants. At the beginning of each interview, the researcher will explain the project, the expected participation, and the associated risks. The interview process and data collection will be explained. There will also be a question period. Following this introduction, written consent will be obtained. All research data (eg interview recordings and transcripts) will be anonymized and stored on a secured server; files will be stored in a password-protected folder on a computer from the Centre de recherche médicale de l'Université de Sherbrooke research chair. All files will be retained for 7 years in accordance with institutional policies. It is also important to note that our study will comply with ethical considerations according to the Tri-Council Policy Statement: Research Ethics (2022) [66].

## Results

The project was funded in March 2022. Data collection activities were completed in November 2024 following the recruitment and interview of participants living with chronic illness who received primary care services via telehealth. The recruitment process met the predefined sampling targets, ensuring adequate representation for qualitative analysis. Data collection was concluded after 22 interviews once thematic saturation was achieved, that is, no new themes or insights emerged from successive interviews, consistent with qualitative methodological guidance [67]. This decision was discussed and validated among the research team. As of December 2025, all participants had been recruited, and the qualitative data analysis was underway. Multiple manuscripts are in development, and the first set of findings was submitted for publication in fall 2025.

## Discussion

### Anticipated Findings

This study will better support and improve IPC in the context of telehealth using a patient-centered approach. Journey mapping will identify potential facilitating factors for improving primary care in telehealth settings, based on the patient’s pathway. Recommendations will be made to build a practical guide supporting IPC in the primary care telehealth context. Various strategies will be implemented by the research team to promote knowledge transfer and knowledge integration.

This research possesses several notable strengths. First, it adopts a patient-centered research approach [40] and strongly collaborates with a patient partner, thereby ensuring the inclusion of perspectives and experiences of individuals directly affected by the health condition under investigation. Second, the use of journey mapping offers a comprehensive understanding of the patient journey, particularly emphasizing patient engagement within care processes of the IPC process, including its facilitators and barriers. This approach will enhance the knowledge transfer process and has the potential to positively influence primary care practice. Furthermore, this study contributes to addressing a gap in the current literature concerning the patient perspective on IPC in the context of telehealth.

Memory bias represents a significant limitation, as the intervention requires participants to recall information that may span 12 months [66]. However, the implementation of the pathway mapping approach serves as a method [46,66] to reengage memories, thereby mitigating this bias [58,59]. Another limitation of this study is that it was carried out only in 2 U-FMGs. This choice was deliberate to ensure consistency in organizational context and access to comparable interprofessional team structures, which supported the study’s analytic depth rather than statistical generalization. We acknowledge that this may limit the generalizability. These teaching institutions are more supervised at the IPC level and better equipped for telehealth [15]. However, the team’s research expertise, having already conducted several qualitative studies with U-FMGs, as well as the approach used, allows us to reduce this bias and truly understand how the U-FMG context may influence the results and transferability.

### Conclusions

This research protocol presents a qualitative study designed to explore the experiences and perspectives of patients living with chronic illness regarding IPC in a telehealth context within primary care. Given the increasing reliance on telehealth modalities to deliver care, particularly for patients with complex and ongoing needs, this study addresses a critical gap in current knowledge. By focusing on the patient perspective, the findings are expected to inform best practices for IPC, guide improvements in telehealth service delivery, and support the development of patient-centered, coordinated care pathways. The results will contribute to optimizing the integration of telehealth in chronic disease management, ultimately enhancing the quality and continuity of care in primary care settings.

## Supplementary material

10.2196/79019 Multimedia Appendix 1 Montreal model.

10.2196/79019 Multimedia Appendix 2 Example of pathway mapping.

10.2196/79019 Multimedia Appendix 3 Interprofessional Education for Collaborative Patient-Centred Practice model.

## Abbreviations

IECPCP: Interprofessional Education for Collaborative Patient-Centred Practice
IPC: interprofessional collaboration
U-FMG: university family medicine group
