# Supplementary figures and images for: Patient Living With Chronic Illness Perception of Interprofessional Collaboration in a Telehealth Context in Primary Care: Protocol for a Qualitative Descriptive Study
Source: JMIR Res Protoc. 2025 Dec 24;14:e79019. doi: 10.2196/79019 (PMC12779463; doi:10.2196/79019)

Additional File 2


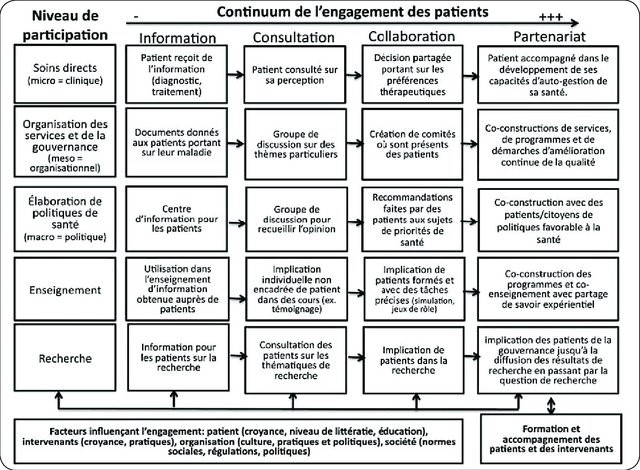

Supplement: Multimedia Appendix 1 [file resprot-v14-e79019-s001.docx]

Additional File 3


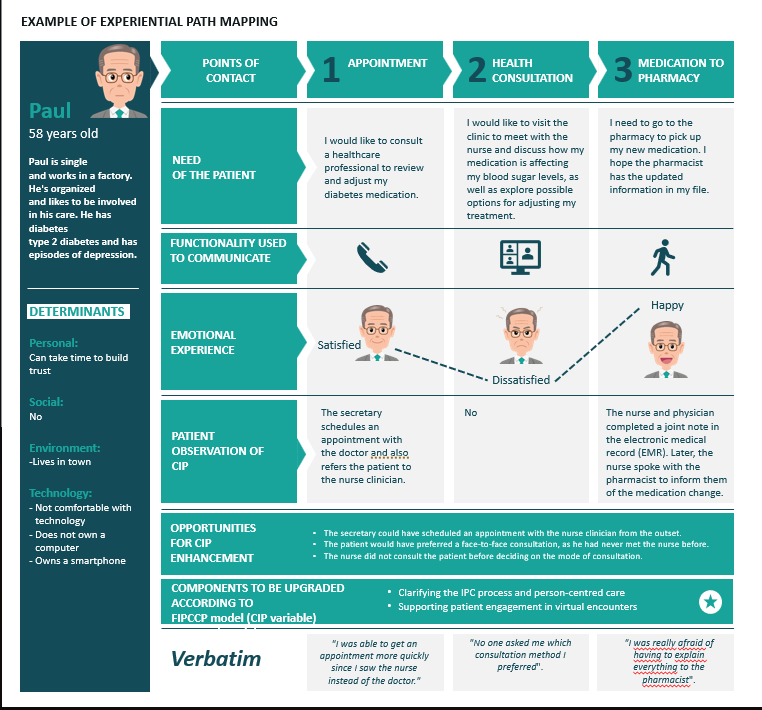

Supplement: Multimedia Appendix 2 [file resprot-v14-e79019-s002.docx]

Additional File 1


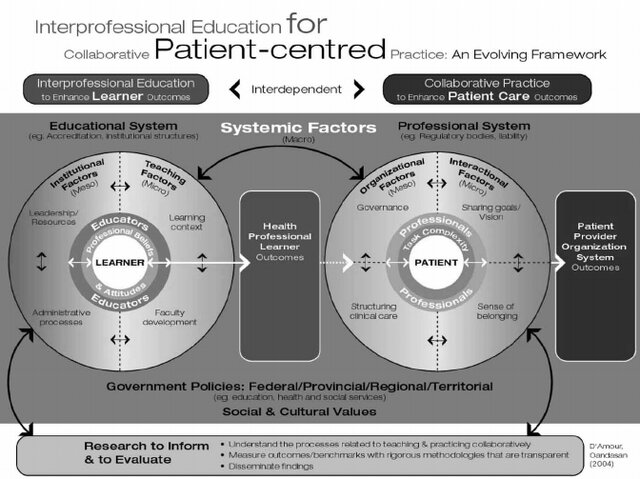


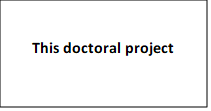

Supplement: Multimedia Appendix 3 [file resprot-v14-e79019-s003.docx]
